# Supplementary figures and images for: Synthetic Data Resource and Benchmarks for Time Cell Analysis and Detection Algorithms
Source: eNeuro. 2023 Mar 15;10(3):ENEURO.0007-22.2023. doi: 10.1523/ENEURO.0007-22.2023 (PMC10027052; doi:10.1523/ENEURO.0007-22.2023)

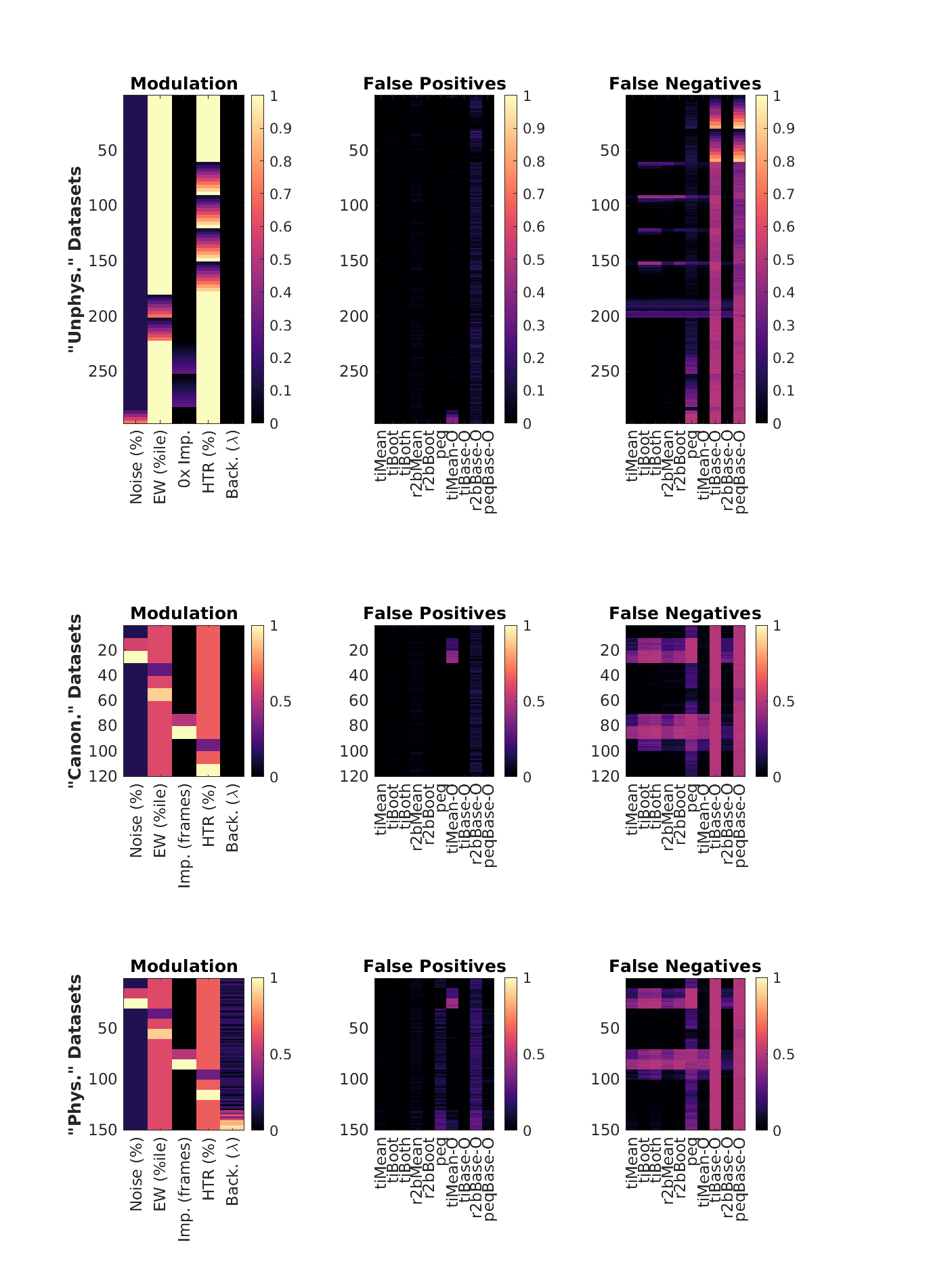

Supplement: Extended Data Figure 1-1 — Modulation profile along with the false positive and false negative rates per dataset, for important parameters configured in each of the 567 synthetic datasets generated. A–C, “Unphysiological Regime.” D–F, “Canonical Regime.” G–I, “Physiological Regime.” Download Figure 1-1, TIF file. [file enu-eN-MNT-0007-22-s04.tif]

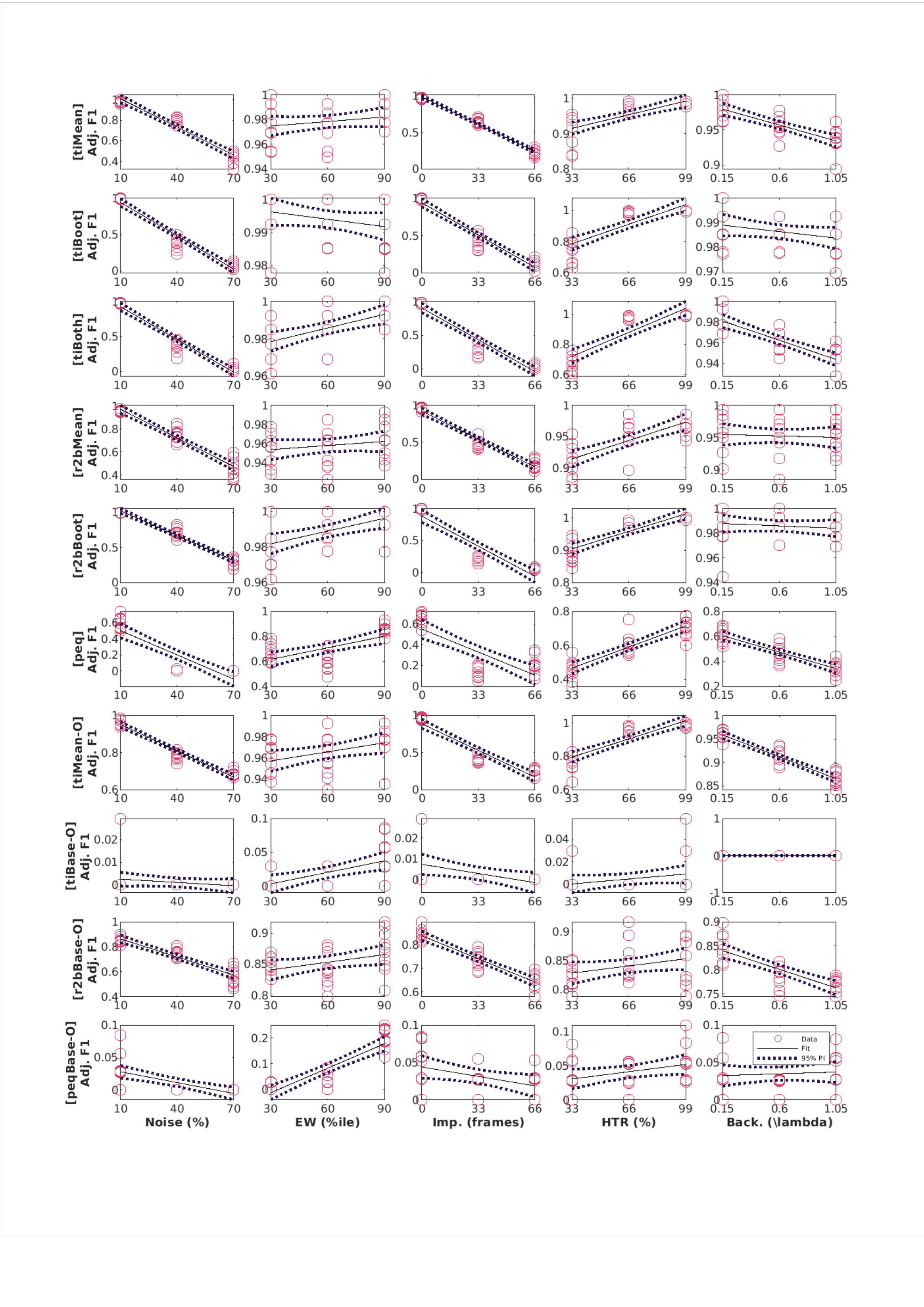

Supplement: Extended Data Figure 6-2 — Linear regression fits for all algorithm parameter dependence curves with data points (red circles), best fit line (black), and the 95% prediction interval (PI; dotted black lines). The columns represent the physiology regime modulation parameter (out of the 5 main parameters tested), and the rows represent the various implemented algorithms for time cell detection. Download Figure 6-2, TIF file. [file enu-eN-MNT-0007-22-s06.tif]
